# Analyses of blood donor samples from eight provinces in Lao PDR suggest considerable variation concerning HBV exposure and carriage

**DOI:** 10.1371/journal.pone.0259814

**Published:** 2021-12-13

**Authors:** Phonethipsavanh Nouanthong, Lisa Hefele, Jerapha Keokhamphue, Vonhphet Sorrasin, Vilaysone Khounvisith, Chanthala Souksakhone, Prapan Jutavijittum, Claude P. Muller, Antony P. Black, Judith M. Hübschen

**Affiliations:** 1 Lao-Lux Laboratory, Institut Pasteur du Laos, Ban Kao-Gnot, Sisattanak District, Vientiane, Lao PDR; 2 Department of Infection and Immunity, Luxembourg Institute of Health, Esch-sur-Alzette, Grand-Duchy of Luxembourg; 3 National Blood Transfusion Center, Lao Red Cross, Rue Phai Nam, Vientiane, Lao PDR; 4 Department of Pathology, Faculty of Medicine, Chiang Mai University, Chiang Mai, Thailand; Ramathibodi Hospital, Mahidol University, THAILAND

## Abstract

**Introduction:**

Hepatitis B is endemic in Lao PDR and about 9% of the adult population is chronically infected. In this study, we investigated regional, occupational, age and sex-related differences in hepatitis B epidemiology in Lao blood donors.

**Methods:**

5017 voluntary blood donors from 8 different provinces were tested for hepatitis B markers by ELISA. Predictors for the prevalence of hepatitis B surface antigen (HBsAg) and antibodies against the core antigen (anti-HBc) were assessed by bivariate and multivariable analyses.

**Results:**

In total, 41% of the participants were positive for anti-HBc; the HBsAg prevalence was estimated at 6.9% among all participants (9.2% among first-time donors and 3.9% among repeat donors). Among first-time donors, HBsAg positivity was associated independently with being male (p<0.001), being from the North (p<0.001) and being soldier (p<0.001). Participants were more likely to be anti-HBc positive when they were male (p<0.001), from the Northern provinces (p<0.001) and older than 20 years (p<0.01).

**Conclusion:**

In conclusion, our study confirmed an overall high HBsAg and anti-HBc prevalence in Lao PDR, albeit with considerable regional variation. The identification of a sizeable number of HBsAg positives among repeat donors warrants a thorough investigation of current blood screening, record keeping, donor identification and counselling practises.

## Introduction

Hepatitis B is a major public health problem worldwide. Chronic hepatitis B virus (HBV) infection causes severe liver damage and 25% of patients die later from related chronic active hepatitis, cirrhosis or primary hepatocellular carcinoma (HCC) [1–3]. Mother-to-child transmission is generally the most common transmission route in Southeast Asia. The risk of developing chronic hepatitis B is as high as 90% if infected at birth and decreases to 5–10% if infection occurs in adulthood [4,5]. Highly endemic countries have chronic HBV infection rates between 8–20%, e.g. parts of Southeast Asia, tropical Africa and parts of China [6–10]. In these regions, the burden of HBV-related HCC and hospitalization are dramatically high.

Hepatitis B vaccination was introduced in Lao PDR as a three-dose course in combination with Diphtheria-Tetanus-Pertussis in 2001. An additional birth dose followed in 2003 [11,12]. Since 2009, the pentavalent diphtheria-tetanus-pertussis-hepatitis B-*Haemophilus influenzae* type b vaccine (DTPw-HepB-Hib) has been used. In 2018, the estimated coverage for the 3^rd^ dose of DTPw-HepB-Hib was 84% [13], while the birth dose coverage was only 55% [14].

More than 40% of the adult Lao population, born before introduction of the vaccine, are likely to have been exposed to the virus [12,15,16]. Despite representing a substantial public health burden, the epidemiology of hepatitis B in Lao PDR is not well understood. The lack of HBV surveillance and limited access to testing undermine efforts to assess HBV treatment and disease burden in the whole country. In recent years, studies investigated the prevalence of chronic infection among mothers and their children as well as protection levels due to vaccination [12,17–20].

In Lao PDR, more than 30,000 blood units are used for transfusion every year (Dr. Chantala Souksakhone, Director of National Blood Transfusion Center, personal communication, 2018). The high endemicity of HBV increases the relative risk of transfusion transmitted infection (TTI). The WHO recommends a mandatory pre-transfusion blood screening for HBV, Hepatitis C Virus (HCV), Human Immunodeficiency Virus (HIV), and Syphilis in all countries [21]. The routine screening for HBV infection was introduced by, the Lao National Blood Centre (NBC) in the early 1970s and includes the screening for the hepatitis B surface antigen (HBsAg) but not for the anti- hepatitis B core antibodies (anti-HBc) due to limited resources. Blood donors positive for HBsAg are informed of their HBV infection status, counselled and advised not to return for blood donation (Dr. Chantala Souksakhone, personal communication, 2018). However, due to challenges with donor compliance, counselling and maintenance of donor records, some HBsAg positive donors still return for blood donation. Furthermore, due to limited resources, the blood is currently screened only for HBsAg, but not for antibodies against HBV. Blood donors are not compensated for their donations.

In this study, we investigated a large cohort of Lao first time and repeat blood donors enrolled at eight different blood donation sites nationwide for markers of HBV infection in order to determine regional, occupational and age or sex related differences.

## Methods

### Participants

From November 2013 to May 2015, we recruited 5017 voluntary blood donors during routine blood donation visits for this study. Recruitment took place at 8 different blood centers: the NBC in Vientiane Capital in Central Lao PDR, Khammouane (KHM) and Attapeu (ATP) in the South, and Luang Prabang (LPB), Luang Namtha (LNT), Phongsaly (PSL), Huaphan (HPN) and Xaiyabuly (XAY) in the North (S1 Fig).

Blood donor eligibility was assessed according to the standards of the National Blood Transfusion Service and included history of blood donations. For donors less than 18 years old, the recommendation is to have school administrators to approve on behalf of the parents and to provide the telephone number of the parents to inform them and get verbal consent. The blood donors were informed about the study and enrolled after written informed consent. According to the regulations of the Lao ethics committee, people above 15 years of age are allowed to provide consent for study participation. The study, including the informed consent form, was approved by the Lao National Ethics Committee for Health Research (reference NECHR 059/2013 and 059/2014). All experiments were done in accordance with relevant regulations and guidelines.

### Sample and data collection

5 ml blood were collected at the time of blood donation. Samples collected by either fixed or mobile units of NBC were shipped to Institut Pasteur du Laos (IPL) for serum separation, storage and subsequent laboratory testing. At the provincial level, the samples were processed at the study site. The serum was frozen at -20°C and shipped to IPL every three months. All serum samples were stored at -80°C at IPL until analysis. The demographic data of donors were collected, including age, sex and occupation, as well as number of donations, date and location of blood donation.

### Laboratory analyses

Commercial ELISA kits (Diasorin, Italy) were used to detect anti-HBc and anti-HBs antibodies. Anti-HBc positive samples that were negative for anti-HBs antibodies or with unknown anti-HBs status, were tested for HBsAg (Diasorin, Italy). Anti-HBc negative samples were considered to be negative for HBsAg. For the purpose of this study, HBsAg positivity was interpreted as chronic infection. Anti-HBc positive results indicated previous exposure to the virus, whereas anti-HBs positive results were indicative of immune protection following infection (in the presence of anti-HBc antibodies) or vaccination (anti-HBs alone). According to the manufacturer’s instruction, a titer ≥ 10 IU/L was considered as reactive for anti-HBs. Samples with an OD value within the +/- 10% range of the OD value of the cut-off calibrator were considered as “equivocal” for anti-HBs.

### Data analyses

Data analyses were conducted using R statistical software [22] with the following packages: tidyverse [23], MASS [24], car [25], lmtest [26] and epitools [27]. For first-time donors, bivariate and multivariable (logistic regression) analyses were performed in order to investigate associations with HBsAg or anti-HBc positive status. In bivariate analyses, odds ratio, 95% confidence intervals (CI) and p values were calculated for seropositive participants. Binary regressions were done with a stepwise method for removing variables not associated with the response variable one by one, considering the p-value of the variable as well as the Akaike Information Criterion (AIC) of the model. A p value <0.05 was considered statistically significant. A correlation was assumed if a correlation value of more than 0.5 or a variance inflation factor of more than 5 was obtained. The decision to exclude a variable was made on a case-by-case basis, with the variable considered as less important and/or with the lower impact being removed. The variables in the models were tested for potential interactions. The final model was compared with the null model using a likelihood ratio test (R function “Anova” with argument test set to “Chisq”). The individual effect of the variables was tested using Wald tests (R function “Anova” with argument test.statistics set to “Wald”).

## Results

### Participant characteristics

From the 5017 participants included, the majority were male (68.1%), students (68.3%) and younger than 25 years of age (80.3%, mean age = 22.2 years, ranging from 16 to 65 years) (S1 Table). Information regarding the frequency of blood donations was available for all but 5 participants. More than half of the 5012 participants were first-time donors (55.8%), 21.1% gave blood for the second time and all others more than two times (ranging from 3 to 35 times).

### Serological profiles of HBV markers

#### Overall serological profiles

All 5017 samples were tested for anti-HBc antibodies and 2056 (41%) were positive (Table 1). Due to limited serum volume and financial constraints and because the focus of the study was on anti-HBc and HBsAg prevalence, only 3602 participants were in addition tested for the presence of anti-HBs antibodies. 20.3% were positive for anti-HBs and 73 (1.5%) participants had an equivocal result. Only 3.5% of all participants were anti-HBs positive and anti-HBc negative, indicating previous vaccination. 636 participants were anti-HBc positive and anti-HBs negative, and from those, 247 were HBsAg positive (4.9% of the total). For 533 anti-HBc positive participants, the anti-HBs status was unknown and 95 of them were HBsAg positive. Due to low volumes of the serum samples, 35 (3%) could not be tested for HBsAg (Table 1). Overall, 342 of the 1134 participants tested were positive for HBsAg. For this study, we considered all anti-HBc negative samples as well as anti-HBc positive and anti-HBs positive or equivocal samples as negative for HBsAg.

**Table 1:** Serological profiles of the participants.

Serological profiles (N = 5017)		n (%)	n HBsAg (+)
anti-HBc (-)	anti-HBs (-)	1874 (37.3)	
	anti-HBs (+)	175 (3.5)	
	anti-HBs (+/-)	30 (0.6)	
	anti-HBs status unknown	882 (17.6)	
anti-HBc (+)	anti-HBs (-)	636 (12.7)	247/629† (39.3%)
	anti-HBs (+)	844 (16.8)	
	anti-HBs (+/-)	43 (0.9)	
	anti-HBs status unknown	533 (10.6)	95/505‡ (18.8%)

Anti-HBc = anti-hepatitis B core antibody; anti-HBs = anti-hepatitis B surface antibody; HBsAg = hepatitis B surface antigen.

† Due to low volume, the serum of 7 participants could not be tested for HBsAg ‡ Due to low volume, the serum of 28 participants could not be tested for HBsAg.

#### Serological profiles by participant characteristics

The proportion of anti-HBc positives was higher in male donors, in participants ≥36 years and in participants from the Northern provinces (S2 Table). Male participants, donors from Northern provinces and soldiers showed a higher prevalence of chronic infection.

**Fig 1:**
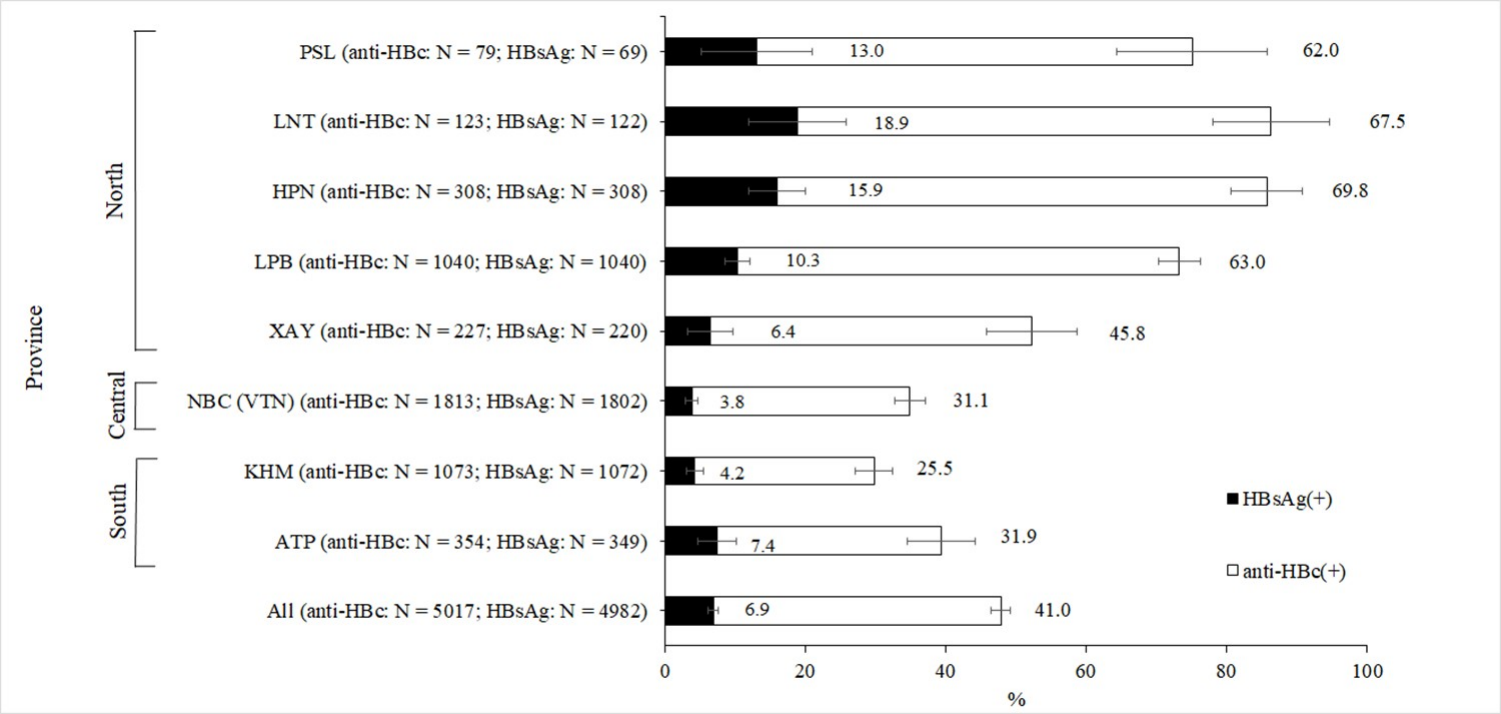
Anti-HBc and HBsAg prevalence by province. PSL = Phongsaly, LNT = Luang Namtha, HPN = Huaphan, LPB = Luang Prabang, XAY = Xayabouli, NBC = National Blood Centre in Vientiane (VTN), KHM = Khammouane, ATP = Attapeu. Error bars represent the 95% confidence interval. Numbers besides the error bars indicate the percentage per province. Numbers in brackets indicate the number of participants tested for anti-HBc and the total numbers taken into account for calculating the HBsAg prevalence. Due to low serum volumes, not all anti-HBc positive samples could be tested for HBsAg.

The prevalence of anti-HBc antibodies and HBsAg varied strongly by geographic location (**Fig 1**, S2 Table). Participants from provinces located in the North of Lao PDR seemed to be disproportionally affected by HBV infection with an anti-HBc seropositivity of 62.2% and HBsAg prevalence of 11.5% as compared to 27.1% and 5.0% in the South. The lowest rate of anti-HBc positivity was found in Khammouane province (25.5%) while the highest rate was observed in participants from Huaphan province (69.8%). The highest rate of chronic infection was observed in Luang Namtha (18.9%).

#### Serological profiles of first-time donors compared to repeat donors

From the 5012 participants with known numbers of blood donations, 55.8% were first-time donors. All 2799 first-time donors were tested for anti-HBc, but due to low sample volumes, only 2778 were tested for HBsAg. The overall anti-HBc prevalence was significantly higher in first-time donors than in repeat donors (44.1% vs. 37%; p<0.001). Anti-HBc seropositivity in first-time donors also varied by province (S3 Table). The prevalence of anti-HBc was higher in the Northern provinces compared to the other provinces irrespective of the age group.

**Fig 2:**
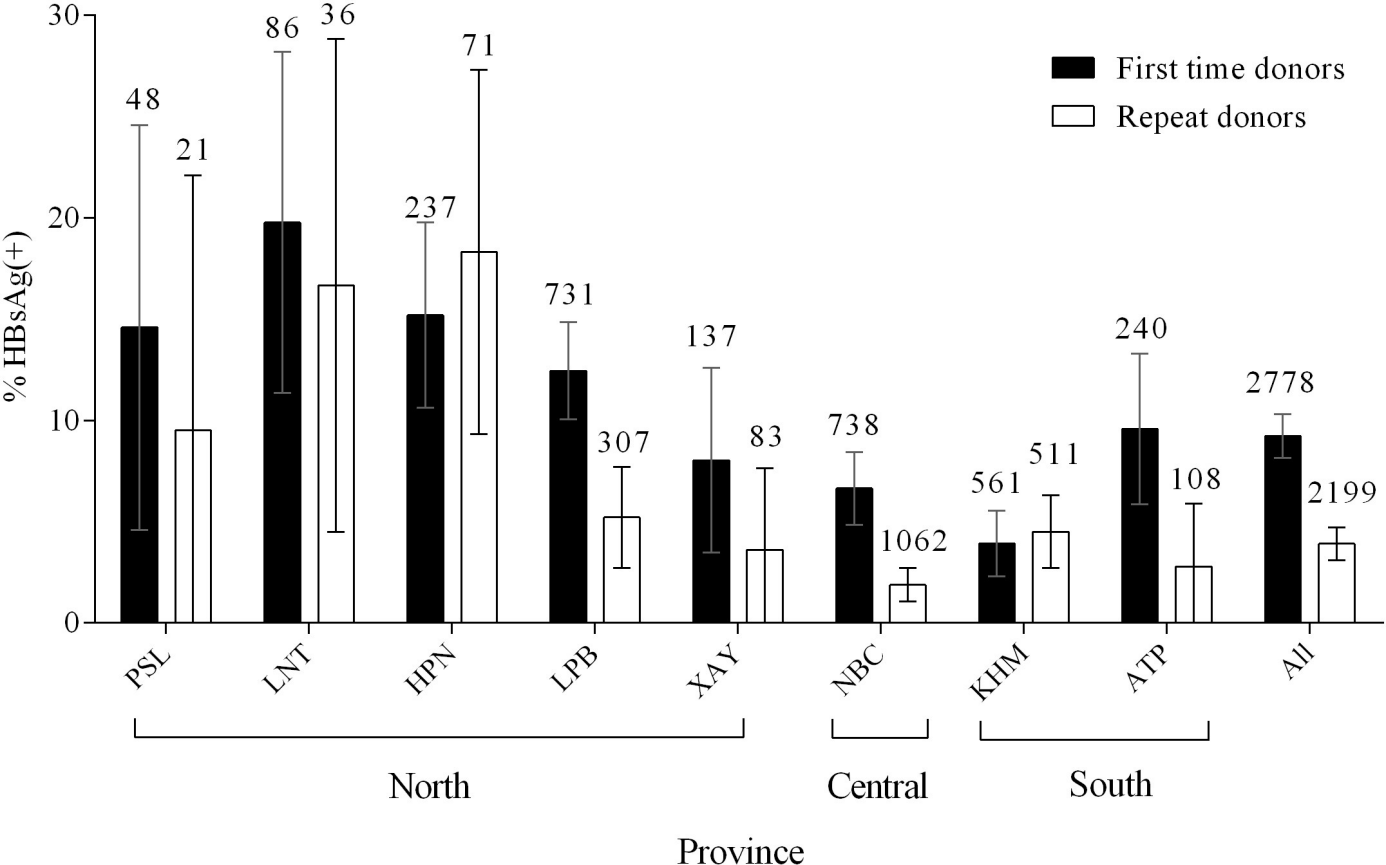
HBsAg prevalence in first-time and repeat donors by province. Error bars represent the 95% confidence interval. Numbers above the error bars indicate the total numbers considered. PSL = Phongsaly, LNT = Luang Namtha, HPN = Huaphan, LPB = Luang Prabang, XAY = Xayabouli, NBC = National Blood Centre in Vientiane, KHM = Khammouane, ATP = Attapeu. Information on frequency of blood donation was not available for 5 individuals.

**Fig 3:**
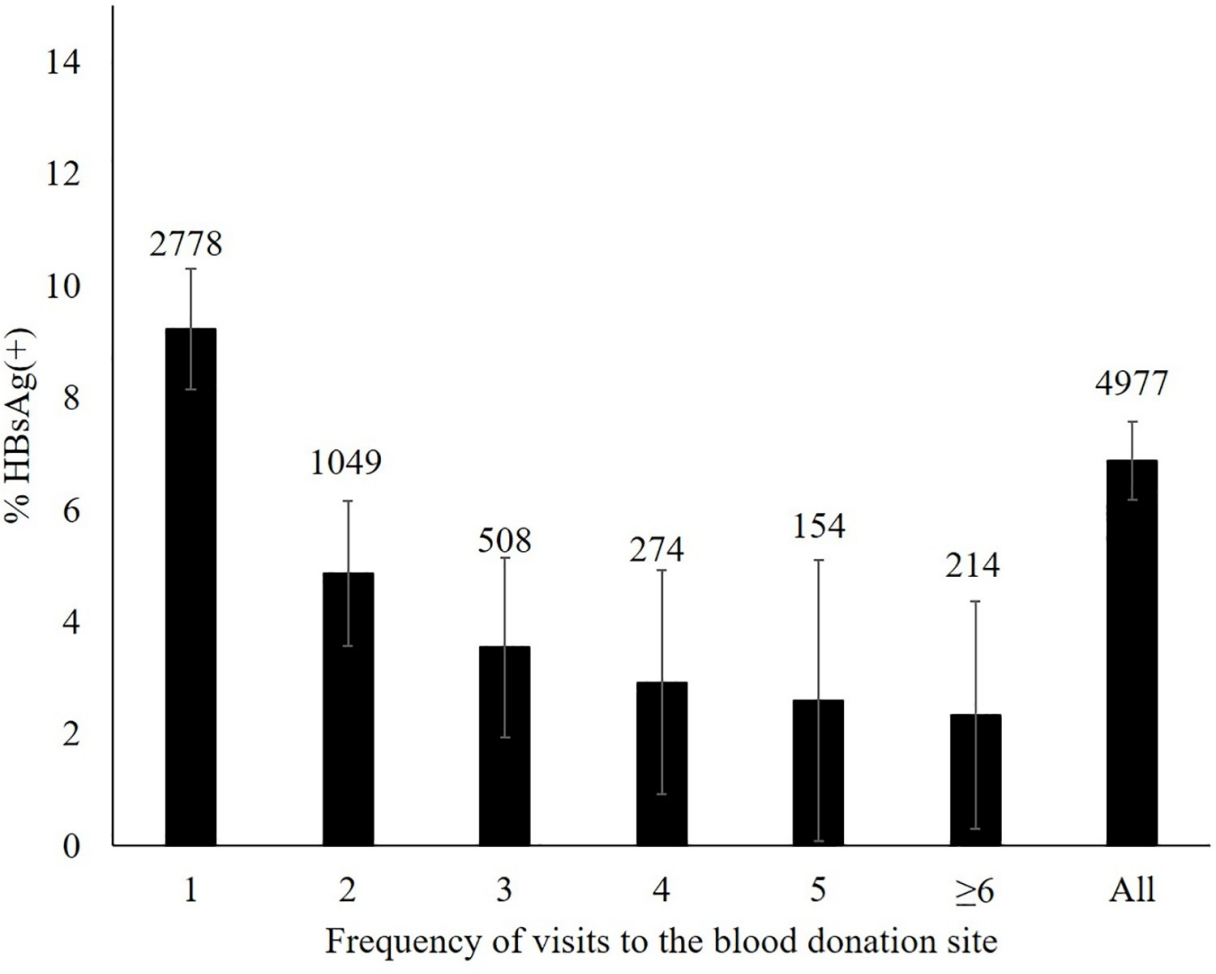
Prevalence of HBsAg by number of blood donations, including the current donation at the time of sample collection. Error bars represent the 95% confidence interval. Numbers above the error bars indicate the total numbers considered. Information on frequency of blood donation was not available for 5 individuals.

Likewise, the prevalence of HBsAg was significantly higher in first-time donors than in repeat donors (9.2 vs. 3.9; p<0.001) and HBsAg prevalence varied by province (**Fig 2**). The prevalence of HBsAg in first-time donors was higher in the Northern provinces (13.1%) compared to Central and Southern provinces (6.6% and 5.6% respectively). The prevalence of HBsAg declined with increasing numbers of blood donations (**Fig 3**).

### Factors associated with HBsAg positivity in first-time donors

After bivariate analysis, all variables were included in the logistic regression model to evaluate the relative contribution of each factor on being seropositive for HBsAg. Three variables remained independently associated with being seropositive for HBsAg. Male participants, soldiers and participants from Northern provinces were significantly more likely to be positive for HBsAg (Table 2).

**Table 2:** Risk factor analysis for HBsAg seroprevalence among first-time donors (N = 2778).

Variable			Bivariate analysis			Multivariable analysis		
		n HBsAg (+)/N total per group (%)	OR	95% CI	P-value	OR	95% CI	P-value
Age (years)	≤ 20	157/1849 (8.5)	Ref					
	21–25	53/480 (11.0)	1.34	[0.96–1.86]	0.089			NS
	26–30	27/222 (12.2)	1.49	[0.97–2.3]	0.080			
	31–35	10/95 (10.5)	1.23	[0.65–2.49]	0.454			
	≥ 36	9/132 (6.8)	0.79	[0.39–1.58]	0.625			
Sex	Female	44/929 (4.7)	Ref					
	Male	212/1849 (11.5)	2.6	[1.86–3.64]	<0.001	2.23	[1.6–3.19]	<0.001
Occupation	Student	162/1947 (8.3)	Ref					
	Office worker	10/186 (5.4)	0.63	[0.32–1.21]	0.200	0.82	[0.39–1.53]	0.560
	Soldier	66/426 (15.5)	2.02	[1.48–2.75]	<0.001	1.65	[1.18–2.3]	<0.001
	Other	18/219 (8.2)	0.99	[0.59–1.64]	1.000	0.7	[0.4–1.15]	0.180
Region	Centre	49/738 (6.6)	Ref					
	South	45/801 (5.6)	0.84	[0.55–1.27]	0.460	0.68	[0.43–1.05]	0.080
	North	162/1239 (13.1)	2.12	[1.52–2.95]	<0.001	1.84	[1.3–2.63]	<0.001

OR = odds ratio; CI = confidence interval.

### Factors associated with anti-HBc positivity in first-time donors

In bivariate analysis, all four variables (sex, age, occupation and recruitment site) were significantly associated with anti-HBc positivity and therefore included in the logistic regression model (Table 3). All variables remained independently associated with being seropositive for anti-HBc. Male participants and participants from the North were 1.5 and 3.3 times more likely to be positive for anti-HBc compared to females and participants from Central provinces. In addition, participants over 20 years of age were more likely to test positive for anti-HBc. In contrast to factors associated with HBsAg positivity, soldiers were not more likely to have been previously exposed to HBV.

**Table 3:** Risk factor analysis for anti-HBc seroprevalence among first-time donors (N = 2799).

Variable			Bivariate analysis			Multivariable analysis		
		n anti-HBc(+)/N total per group (%)	OR	95% CI	P-value	OR	95% CI	P-value
Age (years)	≤ 20	748/1856 (40.3)	ref			ref		
	21–25	237/485 (48.9)	1.42	[1.2–1.7]	<0.001	1.5	[1.2–2.0]	<0.001
	26–30	121/224 (54.0)	1.74	[1.3–2.3]	<0.001	2.2	[1.5–3.2]	<0.001
	31–35	54/100 (54.0)	1.74	[1.2–2.6]	0.009	2.0	[1.2–3.3]	0.006
	≥ 36	73/134 (54.5)	1.77	[1.2–2.5]	0.001	2.3	[1.5–3.7]	<0.001
Sex	Female	334/934 (35.8)	ref			ref		
	Male	899/1865 (48.2)	1.7	[1.7–1.4]	<0.001	1.5	[1.3–1.8]	<0.001
Occupation	Student	804/1958 (41.1)	ref			ref		
	Office worker	60/189 (31.7)	0.7	[0.5–0.9]	0.013	0.5	[0.3–0.8]	0.002
	Soldier	226/432 (52.3)	1.6	[1.3–1.9]	<0.001	0.9	[0.7–1.3]	0.688
	Other	143/220 (65.0)	2.7	[2.0–3.6]	<0.001	0.9	[0.6–1.2]	0.410
Region	Centre	241/743 (32.4)	ref			ref		
	South	210/805 (26.1)	0.7	[0.6–0.9]	0.006	0.7	[0.6–0.9]	0.003
	North	782/1251 (62.5)	3.5	[2.9–4.2]	<0.001	3.3	[2.6–4.0]	<0.001

OR = odds ratio; CI = confidence interval.

## Discussion

In this study, regional, occupation, age and sex-related differences in HBV epidemiology in Lao blood donors were investigated. The seroprevalence of HBsAg extrapolated to 4982 participants was 6.9%, and was 9.2% in first-time donors after excluding repeat donors who showed a much lower proportion of HBsAg positivity (3.9%). This finding is comparable with earlier studies that found an HBsAg prevalence of 8.7% and 9.6% in first-time blood donors [15,16]. In addition, 41% of the participants were positive for anti-HBc, indicating that they had been in contact with the virus at some point during their lives. Virtually all of the donors (>99%) were born before the introduction of HBV vaccination in Lao PDR in 2001, explaining the low seroprevalence (3.5%) of anti-HBs alone profile. Since HBsAg testing was only conducted for anti-HBc positive and anti-HBs negative samples or with unknown anti-HBs status, we cannot exclude that we missed HBsAg positive samples due to false anti-HBs positives or anti-HBc negatives. For the purpose of this study, anti-HBc negative samples were considered negative for HBsAg. These samples were not tested for HBsAg, as only acute infected participants or donors with low infectivity might show this serological profile [16].

Strikingly, the rate of exposure and current infection was much higher in Northern provinces than in the Centre or South of Lao PDR, irrespective of the age group. Such regional variation has not been described before and according to our knowledge, no information about prevalence rates in neighbouring regions of the Northern provinces is available. The observed differences could relate to local variations in risk practices such as for example tattooing, piercings, birth practices and sexual exposure. Indeed, the Khmou, an ethnic group living in the North of Lao PDR, have previously been linked to increased vulnerability to prostitution and increased risk for exposure to HIV or other sexually transmitted diseases [28]. Development work in border areas, such as the improvement of route 3, linking Lao PDR to Thailand and China, has impacted the economic landscape in the Upper Mekong region. Among others, increased prevalence of HIV and rising numbers of small road side shops, often offering alcoholic beverages as well as sexual services, have been cited as negative consequences of this economic shift [28–30]. It has also been reported in other settings that the HBV and hepatitis C prevalence in indigenous populations and ethnic minorities may be higher or different than in the general population [31–36]. Follow-up studies are therefore clearly warranted and should capture potential risk factors as well as ethnicity.

Similar to previous studies [15,16], HBsAg and anti-HBc prevalence rates were higher in males than in females, both in first-time donors and in the overall cohort. The discrepancy could be related to sex-differences in the cellular immune responses to HBV infection [37,38]. This finding should be considered when designing future studies regarding disease burden and exposure.

In comparison to the youngest age group, older participants were more likely to have had an infection with HBV in the past but age did not seem to play a role in the model for being a chronic carrier. This can be explained by the fact that early age infections result more often in chronicity than infections later in life [4,5]. The positive association between age and anti-HBc seropositivity may reflect continuous exposure to hepatitis B infection in an endemic country.

In comparison to students, soldiers were more likely to be chronic carriers but they were not more likely to have had an HBV exposure. Since the high rate of HBsAg positivity indicates early life exposure, it may be hypothesized that soldiers originate more often from less wealthy families with a higher likelihood of HBV exposure than students.

Even though the rate of HBsAg positivity was significantly higher in first-time donors than in repeat donors, the fact that HBsAg positive donors return for blood donation is of concern. There are several possible explanations for this. Firstly, the detection methods used at the Lao Red Cross may not be sensitive enough to detect low levels of HBsAg. This possibility has obvious implications for blood safety and methods for the screening of blood donations should be reviewed. However, it would probably only explain a small proportion of the positive repeat donors. A second possible reason why the repeat donor HBsAg prevalence is high could be that the blood donor database may not be robust enough to exclude returning HBsAg positive donors. In another investigation we have found considerable problems with donor identification across multiple blood donation sites and counselling (manuscript in preparation). Lastly, a small number of the repeat donors may have become infected after the previous donation. Surprisingly and for unknown reasons, the prevalence of HBsAg was higher in repeat donors than in first-time donors in the province Houaphan, a finding which warrants further investigation. The higher prevalence of HBsAg in participants who donated more than 7 times compared to 4 to 6 times can be explained by low overall numbers for each blood donation group.

Serum samples from blood donors are a reasonable surrogate of the general adult population with obvious advantages concerning access and coverage of different geographical regions. A limitation of this study was that, due to financial constraints and low serum volume, not all samples could be tested for anti-HBs. However, since basically all participants were born before vaccine introduction, we do not expect the percentage of donors with post-vaccination serological profile to be much higher than among the tested participants.

Our study investigating more than 5000 blood donors from eight provinces in Lao PDR confirmed an overall high HBsAg and anti-HBc prevalence among first-time donors and for the first time reported considerable regional variations potentially linked to different risk practices. Although the study is focussed on Lao PDR, it showed that important within-country differences regarding HBV exposure may exist, which potentially require tailored public health responses. The identification of a sizeable number of HBsAg positive repeat donors warrants a thorough investigation of current blood screening, record keeping, donor identification and counselling practises to determine which steps of the blood donation process are responsible for this problem. Our findings will hopefully raise awareness also in other resource-limited settings that the monitoring of blood donation procedures may be beneficial to identify and address shortcomings regarding blood donation safety.

## Supporting information

S1 Fig Map of Lao PDR. Provinces included in this study are highlighted in dark grey. PSL = Phongsaly, LNT = Luang Namtha, HPN = Huaphan, LPB = Luang Prabang, XAY = Xayabouli, NBC = National Blood Center in Vientiane (VTN), KHM = Khammouane, ATP = Attapeu. The map was created with QGIS (QGIS Development Team, 2018). The data regarding the administrative boundaries of Lao PDR was obtained from the Humanitarian Data Exchange website (https://data.humdata.org/dataset/lao-admin-boundaries, dataset provided by the National Geographic Department of Lao PDR, 2019) under a CC BY license. Projection used: EPSG 4326 –WGS 84.
(PNG)

S1 Table Participant characteristics by province. (DOCX)

S2 Table Serological profiles according to participant characteristics. (DOCX)

S3 Table Anti-HBc seropositivity by province and age group in first-time blood donors (N = 2799). (DOCX)
