# Supplementary figures and images for: Analyses of blood donor samples from eight provinces in Lao PDR suggest considerable variation concerning HBV exposure and carriage
Source: PLoS One. 2021 Dec 13;16(12):e0259814. doi: 10.1371/journal.pone.0259814 (PMC8668104; doi:10.1371/journal.pone.0259814)

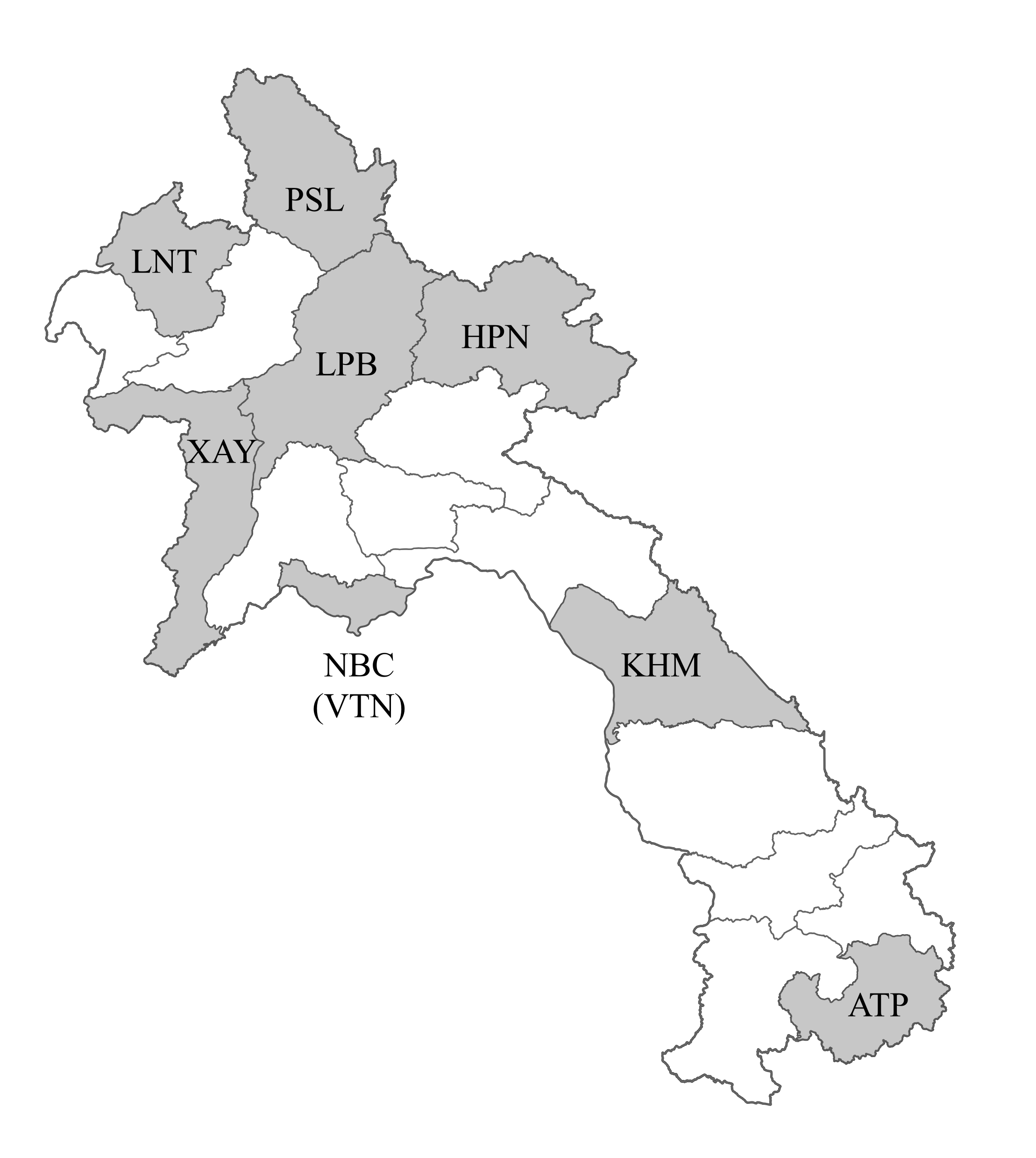

Supplement: S1 Fig — Provinces included in this study are highlighted in dark grey. PSL = Phongsaly, LNT = Luang Namtha, HPN = Huaphan, LPB = Luang Prabang, XAY = Xayabouli, NBC = National Blood Center in Vientiane (VTN), KHM = Khammouane, ATP = Attapeu. The map was created with QGIS (QGIS Development Team, 2018). The data regarding the administrative boundaries of Lao PDR was obtained from the Humanitarian Data Exchange website (https://data.humdata.org/dataset/lao-admin-boundaries, dataset provided by the National Geographic Department of Lao PDR, 2019) under a CC BY license. Projection used: EPSG 4326 –WGS 84. (PNG) [file pone.0259814.s001.png]
