# Supplementary material for: Analyses of blood donor samples from eight provinces in Lao PDR suggest considerable variation concerning HBV exposure and carriage
Source: PLoS One. 2021 Dec 13;16(12):e0259814. doi: 10.1371/journal.pone.0259814 (PMC8668104; doi:10.1371/journal.pone.0259814)
Supplement: S1 Table — (DOCX) [file pone.0259814.s002.docx]

**S1 Table Participant characteristics by province**

|  |  | age group | sex | occupation | | | |  |
| --- | --- | --- | --- | --- | --- | --- | --- | --- |
| province | N | ≤25 years  (%) | male  (%) | student  (%) | office worker (%) | soldier (%) | other  (%) | |
| PSL | 79 | 84.8 | 46.8 | 83.5 | 16.5 | 0.0 | 0.0 | |
| LNT | 123 | 65.9 | 72.4 | 55.3 | 33.3 | 11.4 | 0.0 | |
| HPN | 308 | 93.2 | 68.5 | 71.8 | 2.6 | 25.6 | 0.0 | |
| LPB | 1040 | 75.6 | 72.3 | 58.5 | 2.1 | 19.1 | 20.3 | |
| XAY | 227 | 83.3 | 74.0 | 64.8 | 9.7 | 19.8 | 5.7 | |
| NBC | 1813 | 88.6 | 61.6 | 87.9 | 8.9 | 1.5 | 1.7 | |
| KHM | 1073 | 66.4 | 72.4 | 47.7 | 21.5 | 30.6 | 0.2 | |
| ATP | 354 | 84.5 | 74.9 | 60.2 | 4.8 | 34.7 | 0.3 | |
| total | 5017 | 80.3 | 68.1 | 68.3 | 10.3 | 16.2 | 5.1 | |
| NBC = National Blood Center in Vientiane Capital; KHM = Khammouane; ATP = Attapeu; LPB = Luang Prabang; LNT = Luang Namtha; PSL = Phongsaly; HPN = Houaphan; XAY = Xaiyabuly; N = total number per province | | | | | | | |  |
