# Supplementary material for: Analyses of blood donor samples from eight provinces in Lao PDR suggest considerable variation concerning HBV exposure and carriage
Source: PLoS One. 2021 Dec 13;16(12):e0259814. doi: 10.1371/journal.pone.0259814 (PMC8668104; doi:10.1371/journal.pone.0259814)
Supplement: S2 Table — (DOCX) [file pone.0259814.s003.docx]

**S2 Table Serological profiles according to participant characteristics**

|  |  | **anti-HBc (+) /N group (%)** | **HBsAg (+) /N group (%)** |
| --- | --- | --- | --- |
|  |  |  |  |
| All participants |  | 2056/5017 (40.9) | 342/4982† (6.9) |
|  |  |  |  |
| Age (years) | ≤ 20 | 1108/2992 (37.0) | 195/2980 (6.5) |
|  | 21-25 | 474/1037 (45.7) | 80/1028 (7.8) |
|  | 26-30 | 218/478 (45.6) | 38/474 (8.0) |
|  | 31-35 | 98/208 (47.1) | 13/202 (6.4) |
|  | ≥ 36 | 158/302 (52.3) | 16/298 (5.4) |
|  |  |  |  |
| Sex | Female | 546/1602 (34.1) | 63/1590 (4.0) |
|  | Male | 1510/3415 (44.2) | 279/3392 (8.2) |
|  |  |  |  |
| Occupation | Student | 1310/3429 (38.2) | 209/3412 (6.1) |
|  | Office worker | 191/516 (37.0) | 22/510 (4.3) |
|  | Soldier | 398/815 (48.8) | 93/805 (11.6) |
|  | Other | 157/257 (61.1) | 18/255 (7.1) |
|  |  |  |  |
| Region | Centre | 563/1813 (31.1) | 69/1802 (3.8) |
|  | North | 1106/1778 (62.2) | 202/1759 (11.5) |
|  | South | 387/1427 (27.1) | 71/1421 (5.0) |
|  |  |  |  |
| Anti-HBc = anti-hepatitis B core antibody; HBsAg = hepatitis B surface antigen  †Due to low volume, the serum of 35 anti-HBc positive participants could not be tested for HBsAg; for the purpose of this study all anti-HBc negative samples were considered as negative for HBsAg | | | |
