# Supplementary material for: Analyses of blood donor samples from eight provinces in Lao PDR suggest considerable variation concerning HBV exposure and carriage
Source: PLoS One. 2021 Dec 13;16(12):e0259814. doi: 10.1371/journal.pone.0259814 (PMC8668104; doi:10.1371/journal.pone.0259814)
Supplement: S3 Table — (DOCX) [file pone.0259814.s004.docx]

**S3 Table** Anti-HBc seropositivity by province and age group in first-time blood donors (N = 2799)

|  | **anti-HBc positivity by age group (years)** | | | | |
| --- | --- | --- | --- | --- | --- |
|  | **% (n total of group)** | | | | |
| **Province** | **16-20** | **21-25** | **26-30** | **31-35** | **≥36** |
|  |  |  |  |  |  |
| PSL | 61.7 (47) | 50 (2) | 50 (2) | 50 (2) | 0 (1) |
| LNT | 66 (53) | 55.6 (9) | 66.7 (12) | 60 (5) | 71.4 (7) |
| HPN | 71.5 (186) | 63.6 (44) | 60 (5) | 100 (1) | 100 (1) |
| LPB | 56.1 (412) | 69.2 (130) | 77.3 (88) | 71.7 (46) | 72.7 (55) |
| XAY | 42.7 (110) | 73.7 (19) | 28.6 (7) | 33.3 (3) | 50 (4) |
| NBC | 28.9 (530) | 44.7 (150) | 36.4 (22) | 29.4 (17) | 33.3 (24) |
| KHM | 20.6 (330) | 22.7 (97) | 33.8 (80) | 35 (20) | 42.9 (35) |
| ATP | 27.7 (188) | 29.4 (34) | 50 (8) | 50 (6) | 28.6 (7) |
|  |  |  |  |  |  |
| North (PSL, LNT, HPN, LPB, XAY) | 58.8 (808) | 67.6 (204) | 71.9 (114) | 68.4 (57) | 70.6 (68) |
| Central (NBC) | 28.9 (530) | 44.7 (150) | 36.4 (22) | 29.4 (17) | 33.3 (24) |
| South (KHM, ATP) | 23.2 (518) | 24.4 (131) | 35.2 (88) | 38.5 (26) | 40.5 (42) |
|  |  |  |  |  |  |
| All | 40.3 (1856) | 48.9 (485) | 54.0 (224) | 54.0 (100) | 54.5 (134) |
| NBC = National Blood Center in Vientiane Capital; KHM = Khammouane; ATP = Attapeu; LPB = Luang Prabang; LNT = Luang Namtha; PSL = Phongsaly; HPN = Houaphan; XAY = Xaiyabuly | | | | | |
